# CALFAN (Low *γ*-Glutamyl Transpeptidase (GGT) Cholestasis, Acute Liver Failure, and Neurodegeneration) Syndrome: A Case Report with 3-Year Follow-Up after Liver Transplantation in Early Adulthood

**DOI:** 10.1155/2023/3010131

**Published:** 2023-07-31

**Authors:** Mariam Youssef, Katherine L. Mascia, Brendan McGuire, Chirag R. Patel, Sameer Al Diffalha, Deepti Dhall, Goo Lee

**Affiliations:** ^1^Department of Pathology, The University of Alabama at Birmingham Heersink School of Medicine, Birmingham, AL, USA; ^2^Department of Genetics, The University of Alabama at Birmingham Heersink School of Medicine, Birmingham, AL, USA; ^3^Department of Internal Medicine, The University of Alabama at Birmingham Heersink School of Medicine, Birmingham, AL, USA

## Abstract

CALFAN syndrome is an extremely rare disease consisting of recurrent pediatric acute liver failure (PALF), neurodegenerative diseases, and skeletal abnormalities associated with *SCYL1* gene mutation. To date, three of 18 patients reported underwent liver transplantation in infancy and early childhood (7–23 months). Here, we report a case of CALFAN syndrome with infantile onset, recurrent jaundice/PALF requiring liver transplantation in early adulthood. At the most recent follow-up, 3 years after transplantation, the patient is doing well.

## 1. Introduction

CALFAN syndrome [1, 2], also described as hepatocerebellar neuropathy syndrome [3], is associated with multiple organ involvement including the liver, cerebellum, peripheral nerves, and skeletal system due to *SCYL1* gene mutation [3]. Clinical findings encompass recurrent episodes of PALF/low GGT cholestasis, cerebellar atrophy, ataxia, peripheral neuropathy, short stature, scoliosis, and hip dysplasia. Until now, three cases of liver transplantation have been identified [1, 4] (Table 1) among 18 patients with *SCYL1* mutation reported [1–8].

Here, we report a case of CALFAN syndrome from a 23-year-old-male with recurrent bouts of PALF, hepatosplenomegaly, cerebellar atrophy, and neurocognitive difficulties appeared in his infancy. Liver failure ceased at 2 years of age but hepatosplenomegaly and neurologic symptoms were persistent. The patient was diagnosed with *SCYL1* gene mutation by whole exome sequencing [3] when he was 14. The patient's liver function was well until jaundice recurred at age 20 years. Subsequently, the patient underwent liver transplantation. The patient has been well for 3 years after transplantation.

## 2. Case Report

The patient, a male of white European descent with British and German ancestry, was admitted for evaluation of liver transplantation at age 20 years. Beginning at approximately 9 months of age, he presented with recurrent episodes of PALF. His mother found him icteric after febrile illness. Between these episodes, liver enzymes were normal or exhibited only mild elevations. The patient's liver episodes ceased at 2 years of age but showed hepatosplenomegaly. The very first liver function test (LFT) recorded in our system was at age 12 and demonstrated albumin 5.1 g/dL, total bilirubin (TB) 0.5 mg/dL, AST 36 IU/L, ALT 44 IU/L, alkaline phosphatase (ALP) 207 IU/L, and INR 1.2. Platelet count was 81,000/*μ*L. As per the medical record, the liver biopsy performed at the outside institution at age 4 showed focal bridging fibrosis (slides not available for review).

In addition to the liver issue, his family was concerned about his developmental delay, e.g., started walking at age 2, spoke in several word phrases by age 2-3 years. At age 4, he developed a stutter, which significantly affected his expressive speech. Neurologic symptoms included significant tremors, motor disability, and peripheral neuropathy (e.g., foot drop requiring leg braces). Cavus foot was also noted. In childhood, he developed autistic behavior and attention deficits. A brain MRI at age 9 revealed mild cerebellar vermian atrophy.

Interestingly, one of the patient's sisters had very similar symptoms [3]. No specific etiology was identified despite extensive work-up on both patients including molecular testing for Niemann–Pick C (*NPC1* and *NPC2*), *ATP8B1*, *BSEP*, and *ABCB11* (familial intrahepatic cholestasis); citrin; mitochondrial testing (electron transport chains) and *BCSL1* gene; enzymatic testing including fatty acid oxidation, GM1 gangliosidosis, filipin, Niemann–Pick, gaucher, filipin, fructose 1, 6 bisphosphatase, and aldolase in the liver; biochemical testing including 24-hour urine copper, ceruloplasmin, acylcarnitine, and very-long-chain fatty acid panel; and mutations for cerebellar vermis hypoplasia-oligophrenia-ataxia-coloboma-hepatic fibrosis (COACH) syndrome including MKS3/*TMEM67*, *CC2DA*, and *RPGRIP1L*.

At the age of 14, whole exome sequence analysis [3] revealed to harbor two variants in the *SCYL1* gene, i.e., heterozygous for c.937delG (maternally inherited) and c.1509 1510delTG (paternally inherited) previously unreported [3]. His mother was heterozygote for the c.937delG variant and did not harbor c.1509 1510delTG. His father was heterozygote for the c.1509 1510delTG variant and did not harbor the c.937delG variant. Both parents are asymptomatic. He was well and started a program at age 19 years for kids with intellectual disability for the goal to live independently. His last upper endoscopy was at age 12 with no significant findings. He has had regular abdominal ultrasound examinations that have been stable. His hemoglobin, albumin, and bilirubin at age 19 were within normal range.

**Figure 1:**
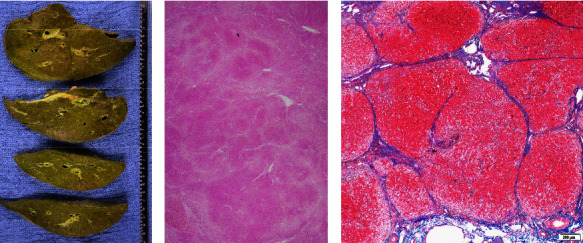
Explanted liver: (a) gross findings. The surface was slightly nodular with green-colored parenchyma. (b) Low magnification H&E image showed nodule formation with fibrous septa, highlighted by trichrome stain (c).

**Figure 2:**
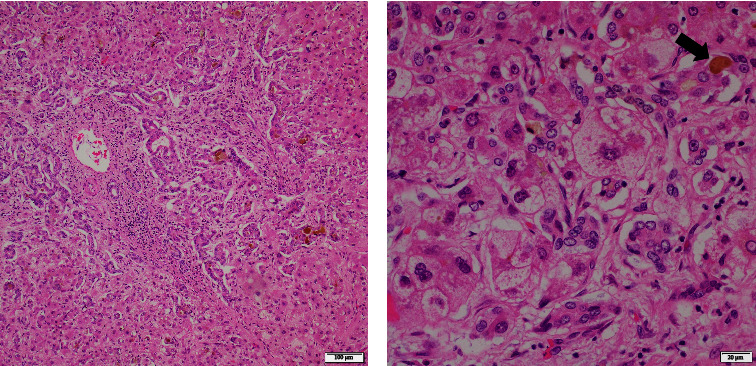
H & E images of the explanted liver: (a) medium magnification view showed a portal tract with extensive bile ductular proliferation. (b) High magnification revealed feathery degeneration of hepatocytes with prominent bile (arrow).

At age 20, he developed jaundice again after fever and rhinorrhea. His LFT was notable for TB 7.7 mg/dL (DB 4.4), ALP 458 IU/L, ALT 692 IU/L, AST 304 IU/L, and INR 2.52. GGT was 20 U/L, consistent with low GGT cholestasis. Platelet count was 33,100/*μ*L. There was no history of acetaminophen or salicylate use. Abdominal ultrasound showed cirrhotic morphology and splenomegaly. He did not respond to conservative management and underwent liver transplantation eventually. The explanted liver demonstrated 1,677 gram weighed green-colored (cholestatic) parenchyma with slightly nodular surface (Figure 1). Microscopic findings showed cirrhosis (Figure 1), cholestatic liver injury, and prominent bile ductular proliferation (Figure 2). Steatosis was minimal.

He recovered well but experienced postoperative biliary stricture requiring several occasions of endoscopic retrograde cholangiopancreatography with stent insertion. After 3 year follow-up, he has been stable with the stricture resolved. He is on tacrolimus and mycophenolate. No clinical suspicion of acute cellular rejection has been identified, nor has posttransplant liver biopsy ever been required. Most recent LFT showed TB 2.1 mg/dL (DB 0.4), ALP 72 IU/L, ALT 18 IU/L, AST 13 IU/L, and GGT 4 U/L. However, he still wears leg braces and has limited expressive language. Follow-up brain MRI also revealed persistent cerebellar atrophy.

## 3. Discussion

*SCYL1* gene encodes SCY1-like-protein 1, a member of the SCY1-like family of pseudokinase involved in intracellular transport processes and regulation of neuronal function and survival [3, 9]. Mechanisms of liver injury remain unclear.

To date, 18 patients of CALFAN syndrome with *SCYL1* mutation including our case have been reported. Most patients showed recurrent hepatopathy with infantile onset, triggered by febrile illness. Low or normal GGT episodes were recorded in 11 of 18 patients. “Bonafide” PALF [10] was documented in 13 of 18 patients but no fatality has been reported. Between episodes LFT and symptoms returned to baseline, however hepatosplenomegaly persisted.

The liver biopsy findings are nonspecific [1, 3–5, 7, 8] including variable degrees of portal/lobular inflammation, fibrosis, cholestasis, steatosis, and giant cell hepatitis. Similar microscopic findings were noted in our case.

Most patients' liver episodes decline or cease in early childhood. Three of 18 patients have been reported to undergo liver transplantation at 23 months of age [1], 7 months [4], and 21 months [4] (Table 1). However, none of the cases were expected to have *SCYL1* mutation at the time of transplantation. No graft failure after transplantation has been reported (follow-up for 8–11 years). Our case was transplanted at 20 years of age while the definitive diagnosis was made at 14 years. Neurodegenerative symptoms appeared to be persistent following liver transplantation, as did our patient's.

In summary, we experienced the first case of CALFAN syndrome requiring liver transplantation in adulthood with the definitive diagnosis. Our patient has been doing well for 3 years following the transplantation without an episode of rejection or significant postoperative complication. As per literature review, graft survival is expected to be excellent.

Except our case, all of transplanted cases were found to have *SCYL1* mutation after transplantation. It is also noted that the liver episodes of CALFAN syndrome appeared to decline with age. Not only is early suspicion of the syndrome with the aforementioned symptom complex important, but also the optimal timing of transplantation should be carefully considered when the diagnosis is made.

## Figures and Tables

**Table 1:** Summary of clinical findings from the patients of CALFAN syndrome with liver transplantation.

Patient	Our case	Lenz et al. [1]	McNiven et al. [4] patient #1	McNiven et al. [4] patient #2
Sex	Male	Female	Male	Male
Age of onset	9 months	6 months	5 months	4 months
Episode of ALF	3	4	4	1
Triggering events of ALF	Fever	Fever, diarrhea	Febrile upper respiratory infection	Lung infection
Hepatosplenomegaly	Yes	Yes	Yes	Yes
Low or normal GGT	Yes (20 U/L)	Yes (38–97 U/L)	Mildly elevated (58–83 U/L)	Yes (32 U/L)
Liver biopsy before transplantation	*At age 4 years*: bridging fibrosis	*At age 6 months*: cholestasis, hepatocyte degeneration, and giant cell transformation	*At age 5 months*: cholestasis, hepatocyte feathery degeneration, and bridging septa	*At age 5 months*: cholestasis
		*At age 13 months*: suggesting cirrhosis, hepatocellular injury, and nonspecific cholangitis		
		*At age 23 month*: stage 3-4 bridging fibrosis with focal nodularity		
Motor/sensory dysfunction, developmental delay, skeletal abnormality	Yes	Yes	Yes	Yes
Age at final diagnosis	14 years	Not recorded	13 years	9 years
Age at transplantation	20 years	23 months	21 months	7 months
Follow up (years) after transplantation and course	3 years; overall excellent; no episode of rejection; postop biliary stricture- recovered	8 years; satisfactory (no other descriptions)	11 years; no episode of rejection; no postop complication	9 years; no episode of rejection; postop incisional hernia and CMV colitis

## Data Availability

The patient data used to support the findings of this study are included within the article.
